# A Survey on the Design of Virtual Reality Interaction Interfaces

**DOI:** 10.3390/s24196204

**Published:** 2024-09-25

**Authors:** Meng-Xi Chen, Huicong Hu, Ruiqi Yao, Longhu Qiu, Dongxu Li

**Affiliations:** Cheung Kong School of Art and Design, Shantou University, Shantou 515063, China

**Keywords:** virtual reality, interaction interface, user experience, design evaluation

## Abstract

Virtual reality (VR) technology has made remarkable progress in recent years and will be widely used in the future. As a bridge for information exchanges between users and VR systems, the interaction interface is pivotal for providing users with a good experience and has emerged as a key research focus. In this review, we conducted a comprehensive search of the Web of Science and CNKI databases from 2011 to 2023 to identify articles dedicated to VR interaction interface design. Through a meticulous analysis of 438 articles, this paper offers a substantial contribution to the emerging field of VR interactive interface research, providing an in-depth review of the principal research advancements. This review revealed that the majority of studies are centered on practical case analyses within specific application scenarios, employing empirical evaluation methods to assess objective or subjective metrics. We then concentrated on elucidating the foundational principles of interface design and their evaluation methodologies, providing a reference for future research endeavors. Additionally, the limitations, challenges, and future directions in VR interaction interface design research were discussed, highlighting the need for further research in design evaluation to continuously refine the development of standards and guidelines for VR interactive interface design. According to the findings of this review, there is a necessity to enhance research on information design for multi-channel interactive interfaces. Furthermore, it is essential to focus on the diverse characteristics of users to propose more inclusive design solutions. Adopting interdisciplinary approaches could lead to breakthroughs in the creation of personalized and adaptive VR interaction interfaces.

## 1. Introduction

Virtual reality (VR) technology creates immersive three-dimensional virtual environments, allowing users to interact with a variety of sensory-rich elements such as virtual objects, tools, and characters [1]. Rapid advancements in VR-related technologies such as computer graphics have facilitated the provision of high-resolution head-mounted displays (HMD), highly sensitive sensors, and low-latency input devices, thereby enhancing users’ immersive and seamless interactive experiences. With the explosion of the “metaverse” concept, there is a growing demand for virtual libraries, virtual museums, virtual concerts, and virtual social interactions. Even in constrained time and space, individuals can freely explore worlds that are difficult to experience in real life through VR devices.

VR exhibits three interrelated characteristics: interaction, immersion, and imagination [2]. The degree of immersion and interaction intensity with virtual elements directly influence users’ imaginations of the virtual environment. Based on immersion levels, VR systems are categorized into non-immersive VR, semi-immersive VR, and fully immersive VR, each employing different combinations of interaction techniques, devices, and interfaces [3]. Non-immersive VR enables interaction with virtual environments on desktop or handheld screens via touch, a mouse, and gestures [4,5,6]. Semi-immersive VR typically consists of a large screen, system, and monitors akin to a cinema experience, often without posture tracking when used by multiple individuals. Andujar et al. [7] provided an example with their virtual viewer designed for cultural heritage exhibitions, where users interact via a desktop interface to view architectural heritage on a projection screen. Fully immersive VR utilizes HMDs or CAVE systems for interaction through multiple devices and channels to maintain immersion [8,9,10,11,12,13,14]. Fully immersive VR is increasingly favored, with studies indicating more accurate spatial perception [15], improved task performance [16], and higher subjective ratings [14,17]. However, some scholars argue that users complete specific tasks faster in non-immersive VR using more familiar interaction devices [18].

**Figure 1:**
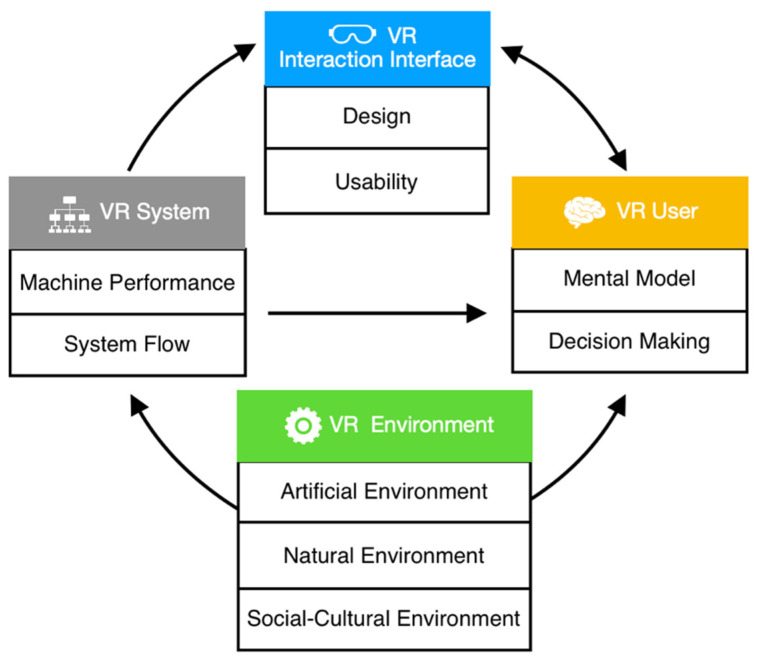
Human-computer interaction in VR.

Human-computer interaction (HCI) is an interdisciplinary field that integrates computer science, psychology, design, information management, sociology, and engineering. We illustrated the relationship between VR HCI as shown in Figure 1, where users interact with the VR interfaces, perceiving and responding in a bidirectional manner. Design elements and the usability of the interaction interface influence users’ mental models and decisions, while user needs and experiences serve as important design criteria. Additionally, users are affected by the operation process, performance, and usage environment of VR systems. Conversely, users can also choose to use artificial, natural, and socio-cultural environments within VR, such as networks, lighting, noise, and customs. Changes in the usage environment result in varying system performance and operation processes, thereby affecting VR interaction interfaces and users. Therefore, it is evident that VR interaction effectiveness is influenced by multiple factors, necessitating the precise delineation of conditions during the design and evaluation of interaction interfaces.

The fundamental issues in VR interaction design research include interaction interfaces, interaction technologies, interaction devices, and task analysis [19]. Generally, VR interaction interfaces consist of multi-touch interfaces, tangible user interfaces (TUIs), three-dimensional (3D) UIs, multi-channel UIs, and mixed UIs. Interaction technologies encompass 3D interaction techniques, gesture recognition, voice recognition and sound interaction technologies, haptic feedback technologies, eye-tracking technologies, pen interaction techniques, and physiological computing techniques [20]. VR systems typically utilize input and output devices such as tablets, PCs, smartphones, interactive desktops, pens and drawing tablets, HMDs, projectors, and physical objects.

For the analysis of VR interaction tasks, studies commonly adopt the three task types proposed by Bowman et al. [21]: selection/manipulation, navigation, and system control. Among these, selection/manipulation tasks have garnered significant scholarly attention, with some studies focusing on the selection or rotation of virtual objects [10,16,22,23,24,25,26,27,28,29]. Navigation tasks involve travel and wayfinding. For example, Lahav [30] invited 15 blind users to explore and construct cognitive maps and perform orientation tasks using the VR system. Sorger et al. [31] required users to traverse a designated path in a 3D space based on HMDs using various interfaces. Sun et al. [32] suggested that the fishing mode, which features instantaneous movement, offers higher learning performance than the flying mode in the UI design for navigation within VR-based architectural applications. Chen and Chen [4] recruited 56 users to complete 3 pathfinding tasks using an overview map in a virtual environment, exploring the impact of design differences in overview maps on pathfinding performance and subjective experience. System control tasks include changing system states and interaction modes. Wang et al. [8] evaluated 3 interaction technologies based on the objective performance and subjective evaluations of 24 users operating fixed or handheld menus in an HMD-based VR environment, ultimately proposing design guidelines for menu interfaces. Zhou et al. [33,34] proposed the use of VR to optimize the control method for smart home interfaces, which addresses their interaction deficiencies, simplifies the control process, and significantly cuts down costs. VR interaction experiences are influenced by technology, devices, and task types, necessitating targeted consideration in design research.

HCI design plays a significant role in enhancing user experiences. Research on interaction design based on traditional graphic user interfaces (GUIs) is no longer sufficient to meet the emerging needs of VR interactions. However, to date, there are no established evaluation standards or systematic design guidelines for VR interaction interfaces. Moreover, the existing work exhibits numerous limitations in VR interaction interface design. For instance, some case studies lack empirical evaluations of different designs of VR interaction interfaces based on specific interaction tasks. Previous research has rarely addressed how VR interaction interface design impacts users with diverse characteristics, and the quantity, diversity, and duration of user studies are quite limited. There has been a proliferation of research on VR hardware and platform development globally. However, a substantial amount of research is framed within the confines of information science and engineering, with few studies adopting an interdisciplinary perspective that integrates knowledge from the humanities and social sciences, such as art, anthropology, and psychology. An increasing number of new types of multi-channel interactive interfaces are being rapidly developed, yet research integrating multiple sensory channels, including vision, hearing, touch, taste, and smell, remains limited. There is an urgent need to understand how users acquire useful information through different senses to subsequently enhance the VR interaction experience. In summary, research on VR interaction interface design is still in its early stages, with a notable absence of comprehensive literature reviews.

This review synthesized the academic research of VR interaction interfaces from 2011 to 2023, with the main objectives of: (1) providing a comprehensive overview of the current state of VR interaction interface design research; (2) exploring existing principles and summarizing evaluation methods for VR interaction interface design; and (3) identifying key issues and charting future research directions for VR interaction interface design. Through a meticulous analysis of the literature, this review endeavors to bridge gaps in knowledge, discern trends, and distill effective practices, thereby informing the evolution of more efficacious design strategies and evaluative techniques for VR interactions.

## 2. Materials and Methods

### Literature Review and Selection Process

In the preparatory phase of this review, we first clarified the basic features of VR interaction and the research questions in order to define the needs and objectives for the current research. Then, we conducted a comprehensive literature review of recent academic achievements in VR interaction interfaces, identified the current research focus, and suggested future research directions.

This review utilized two electronic databases: Web of Science (webofknowledge.com) (Clarivate Analytics, London, UK) and the China National Knowledge Infrastructure (CNKI) Academic Journal Database (kns.cnki.net) (Tongfang Knowledge Network Technology Co. Ltd., Beijing, China). The Web of Science hosts over 12,000 high-quality academic journals, encompassing the Science Citation Index (SCI), Social Sciences Citation Index (SSCI), and Arts and Humanities Citation Index (A&HCI). The CNKI Academic Journal Database includes more than 8570 Chinese academic journals, featuring the Chinese Social Sciences Citation Index (CSSCI), journals listed in Peking University’s “A Guide to the Core Journal of China”, and selected journals indexed in the Engineering Index (EI). Integrating searches across these authoritative databases enabled a comprehensive overview of the research in this field, providing insights into cutting-edge research achievements.

**Figure 2:**
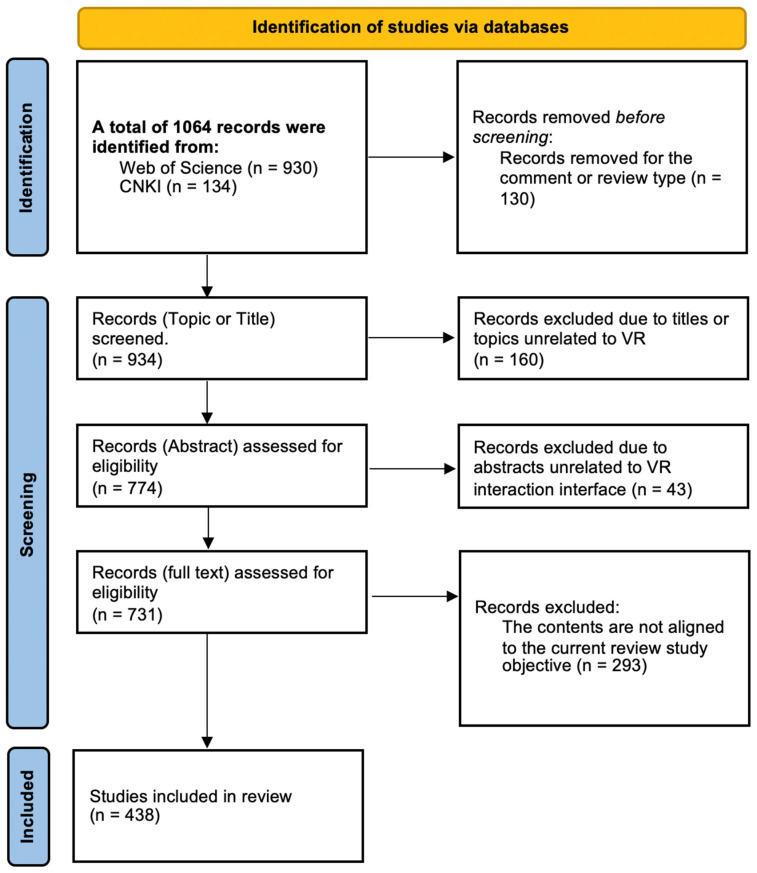
Flow diagram of literature search.

The process of collecting and selecting literature is illustrated in Figure 2. Initially, we conducted a thorough search in both databases for publications with “virtual reality” OR “VR” AND “interaction” AND “interface” in the title, keywords, or abstract. Due to the rapid development of VR interaction technology, many early technologies are no longer in use; hence, only articles published between 2011 and 2023 were collected. We included studies written in both English and Chinese regardless of the authors’ regions of origin. Then, we identified relevant studies through a multi-stage screening process. A detailed screening of the documents was performed, examining the titles, keywords, and abstracts. Articles with non-specific abstracts required full-text review. This review focused on VR interaction interface design, thus excluding unrelated topics such as interaction and modeling technologies and algorithms. Only original research published in academic journals was included, excluding reviews, comments, and commercial articles. Finally, we compiled the selected literature in tables, recording basic information such as the article title, author, source title, document type, keywords, abstract, address, and publication year. The full texts were reviewed to discern the objectives, methods, and conclusions of each study. This examination enabled an evaluation of whether the included studies effectively respond to the initial research inquiry, the suitability of the methods for addressing the research question, and the proper execution of the studies, thereby assessing the overall quality of the research.

The initial search in the Web of Science and CNKI databases yielded 1064 articles, comprising 930 from the Web of Science and 134 relevant articles from the CNKI. A total of 130 commentaries or reviews were excluded due to document type mismatches. During the screening stage, 160 articles with titles or topics unrelated to VR, such as those on augmented reality (AR), material interactions, and social interactions, were excluded. After the assessment of abstracts, 43 articles related to VR design patent protection, VR game structure design, user behavior, and psychological studies were excluded as they did not apply to VR interaction interfaces. Finally, after a detailed examination of the full texts, 293 articles were excluded as they focused on VR interaction technology, systems, and device development, not aligning with the objectives of this review. Following these screening processes, a total of 438 articles were included in this review, comprising 350 English articles and 88 Chinese articles.

## 3. Results and Analysis

Based on the classification and organization of articles and the research purpose of this review, an overview of all selected articles is provided, followed by a detailed analysis of the research subjects and main findings, aiming to reveal the current development of VR interaction interface design research. Subsequently, the design principles and assessment methods widely used in VR interaction interface design research are introduced. Finally, research gaps in these publications are reported, with the aim of providing readers with new research perspectives.

### The Current Development of VR Interaction Interface Design Research

**Figure 3:**
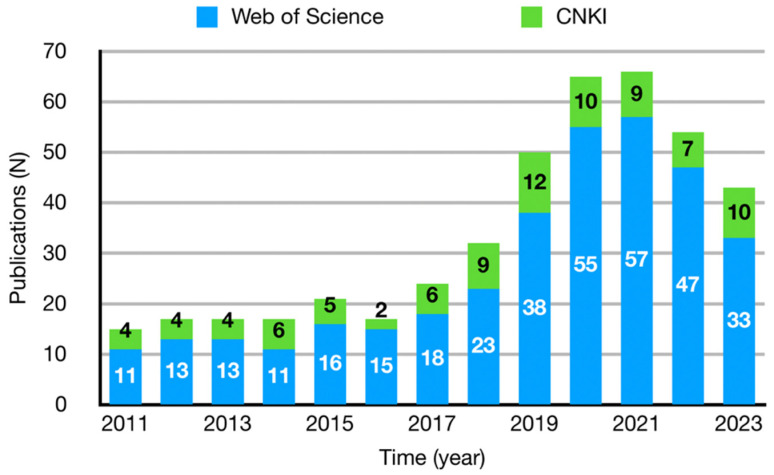
Numbers of publications on VR interaction interface design (from 2011 to 2023).

The number of publications discussing VR interaction interface design published from 2011 to 2023 is shown in Figure 3. The early quantity remained relatively stable, possibly due to limited consumer market penetration of VR interaction technology and expensive devices. With the increasing popularity of technology and devices, the number of related articles published has been accelerating since 2016, but the growth rate has slowed after 2021.

As shown in Table 1, journals that frequently publish literature on VR interaction interface design include “Virtual Reality”, followed by “IEEE Transactions on Visualization and Computer Graphics”, “Applied Sciences—Basel”, “IEEE Access”, and “Multimedia Tools and Applications”. These journals are primarily published in the United Kingdom, the United States, Switzerland, the Netherlands, and China.

**Figure 4:**
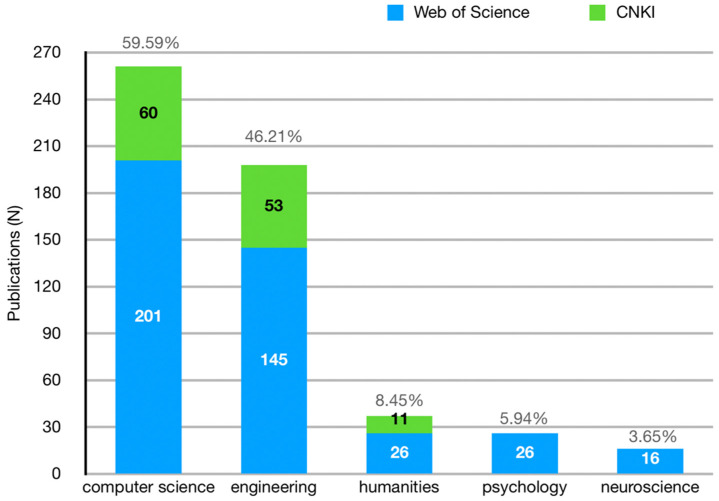
The primary subject distribution in the literature.

Adhering to the database’s official classification system, we performed further analysis on the subject classifications of the articles. We recognized that the database could assign a single article to multiple categories, and this analysis is presented in Figure 4. Computer science is the leading field with 59.59%, followed by engineering at 45.21%. Additional categories include humanities (8.45%), psychology (5.94%), and neuroscience (3.65%). In the humanities, educational research predominates with 18 articles, while a smaller number delves into fields such as management science, museums, and art studies. A significant amount of research integrates interdisciplinary approaches, as evidenced by 67 articles categorized under Multidisciplinary in the Web of Science.

**Figure 5:**
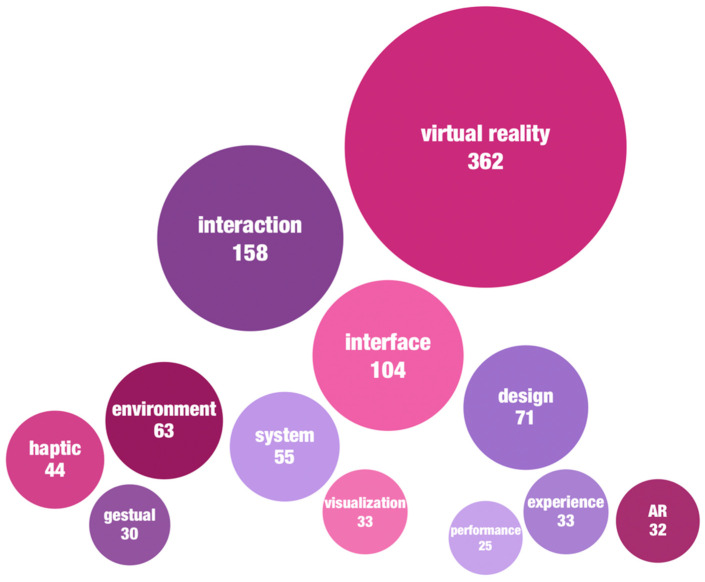
The high frequency keywords in the literature.

To discern the focal points of current research, we analyzed the frequency of keywords in the articles. As shown in Figure 5, the top three most frequently occurring keywords, aligning with the search terms, are “virtual reality” (362 occurrences), “interaction” (158 occurrences), and “interface” (104 occurrences). Following these are “design”, “environment”, “system”, and “haptic”, each appearing more than 40 times. This indicates a general emphasis on the design of interactive environments and systems. Natural HCI is a significant future direction, with haptic- [28,35,36,37,38,39,40,41,42,43,44,45], gesture- [13,14,16,29,46,47,48,49,50,51,52], and posture- [14,53,54]-based interactions gaining considerable attention in VR. For instance, Otaran et al. [36] proposed an impedance-type ankle tactile interface to enhance users’ immersive navigation experiences in VR. Cardoso and Ribeiro [37] designed a touchable TUI for VR books using head-mounted displays (HMDs), aiming to create memorable interaction experiences. Lee et al. [14] developed a posture-based navigation interface for HMDs, allowing users to explore 3D maps by mimicking bird flight postures, thereby enhancing immersion and engagement. Vosinakis and Koutsabasis [16] compared and evaluated visual feedback designs for bare hand interaction (BHI) in VR across five grasp-and-release tasks. The visualization of information in VR is another hot research topic [17,55,56,57,58,59,60]. Sun et al. [61] proposed a distributed cognition-based information visualization model that adjusts resource allocation based on user behavior to reduce cognitive load.

In addition, some studies focus on mixed reality systems, which typically integrate various interaction devices to allow users to perceive information from the physical and virtual environments through multiple sensory channels [11,51,62,63,64,65,66,67,68,69,70]. For example, Zhang et al. [11] physically integrated a VR controller and a smartphone to create a hybrid 2D-3D tangible interface for VR interactions, combining the strengths of both devices. Cho et al. [63] introduced an asymmetric virtual environment supporting non-immersive and immersive participatory modes, integrating personal computers, mobile devices, VR, AR, and motion capture technologies. They conducted statistical analyses to validate user satisfaction with interfaces and their sense of presence in virtual environments. Zhu et al. [64] addressed exploratory experimental teaching needs using formative assessment, proposing a mixed reality experimental system framework tailored to inquiry-based learning theories, exemplified by the development of chemistry experiments and interactive kits. Wang et al. [65] conducted user studies on a mixed VR and AR remote collaboration system, finding that providing tactile feedback through TUI significantly enhances remote experts’ VR interaction experiences.

**Figure 6:**
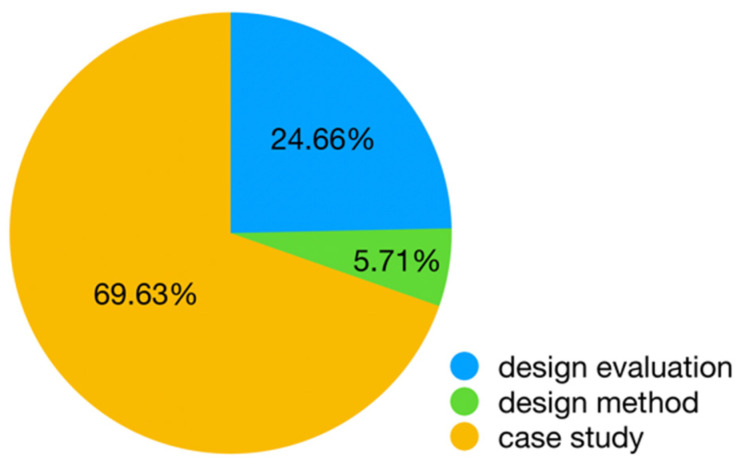
Research topic distribution in the literature.

Among the 438 articles, approximately 69.63% focused on case studies of VR applications in education, industry, healthcare, gaming, exhibitions, and shopping, with a majority in education (102 articles) [32,56,60,71,72,73,74], industry (89 articles) [40,44,51,67,70,75,76,77,78], and healthcare (55 articles) [33,41,54,58,68,69,79,80]. Some applied studies, after a detailed analysis of case designs, also summarize targeted design guidelines through user studies. The rest of the research shifts focus from specific application cases to discuss common issues across various VR applications, evaluating and comparing different interaction interface designs (accounting for approximately 24.66%) [11,35,48,81,82] or proposing more universally applicable design methodologies (about 5.71%) [66,83] (see Figure 6). In the articles retrieved from the Web of Science, the number of articles conducting design evaluations (101 articles) far exceeded those proposing design methods (13 articles). However, in articles retrieved from CNKI, the number of articles proposing design methods (12 articles) surpassed those conducting design evaluations (7 articles). Overall, current research on VR interaction interface design focuses largely on practical applications in specific scenarios, lacking in-depth theoretical exploration. Research providing design guidelines for VR interaction interfaces across different scenarios still requires further development.

## 4. Discussion

### 4.1. VR Interactive Interface Design Principles

In the paradigm of VR interaction, users input commands to the system through devices such as a mouse, a keyboard, a game controller, a touchscreen, as well as more natural methods like eye tracking, speech, gestures, posture, facial expressions, and brainwaves, and perceive feedback from the virtual environment through multiple sensory channels (vision, hearing, touch, force, smell, and taste). The design of VR interaction interfaces requires consideration of various input components, output displays, and interaction metaphors [19], making it difficult to propose universal design principles. Traditional GUI design principles and early VR device design research results should not be directly applied to new VR interaction interfaces. For instance, studies have shown that map size and transparency significantly affect users’ navigation performance and subjective experience in desktop virtual environments [4]. Whether these map design guidelines apply when using wider field-of-view immersive HMDs still needs testing and validation.

Some scholars have attempted to summarize specific perspectives. For example, Zhang et al. [84] proposed that VR interaction interfaces for young people should embody fun, guidance, and emotion through the spatial sense, sound, navigation, layout, and icon design. Li et al. [85] systematically proposed design guidelines for VR teaching applications from the aspects of methods, skills, knowledge, and inspiration, emphasizing the clear display of menu information, user position, learning progress, and operational feedback in teaching scenarios, focusing on customization, fault tolerance, consistency, and naturalness. Qi et al. [86] proposed a multi-modal interaction interface model for digital collection resources, analyzing the user interaction cognitive process of collection resources in VR situations and identifying key factors influencing user experience. Lehrer et al. [87] summarized feedback design guidelines for rehabilitation interaction systems, applied in a VR and AR mixed stroke rehabilitation system. Due to the specificity of research conditions and scenarios, these results cannot be directly used to guide general VR interaction interface design.

Jacob et al. [88] proposed a unified concept: reality-based interaction (RBI), which helps understand, relate, and compare various new VR interaction interfaces, providing important guidance for the design and analysis of interaction metaphors. The RBI framework consists of four themes from the real world, including users’ perception, manipulation, and interaction skills regarding the physical world, their own bodies, the environment, and other people in society. Emulating realistic interaction interfaces may enhance presence and immersion and be more familiar to users, accelerating learning to use VR systems and reducing cognitive load for task processing. However, excessive irrelevant information in the interaction may cause user distraction and lead to comprehension confusion. VR interaction interface design needs to balance realism with usability, functionality, stability, and flexibility.

Furthermore, research findings from psychology and human factors engineering can guide the design of VR interaction interfaces. Human factors engineering takes into account physiological, psychological, and social factors to optimize the relationship between VR systems and users. For example, scholars have summarized methods for quantitatively assessing HMD visual fatigue, such as eye movement, blinking, and regular eye health checks [89]. Principles from visual cognitive psychology, such as attention, memory, and learning, provide fundamental principles for interaction design. Lin et al. [90] created a hybrid VR and AR mobile guidance system based on mental model theory, continuously approaching users’ psychological expectations through repeated usability testing and iterative design. Additionally, many psychological experimental results conducted in VR environments can be directly used to analyze the psychological factors of VR users. For example, psychological experiments on spatial cognition in VR study the underlying mechanisms of human spatial navigation, understanding the establishment, extraction, and transformation of spatial reference frames. This helps explain why VR users have difficulty judging spatial relationships, are prone to spatial anxiety, and make navigation errors, thus establishing VR interaction interface design principles based on spatial cognition to reduce user confusion and frustration.

In summary, the design of VR interaction interfaces should integrate cognitive processes, emotional experiences, multimodal interactions, and principles from psychology and human factors engineering to enhance user experience. The continuous development and improvement can be approached from the following aspects:

(1) Applying cognitive load theory to minimize extraneous cognitive load and enhance germane load, thereby optimizing interface design for reduced cognitive demands during virtual environment interpretation. Adhering to design principles like proximity and consistency can improve users’ understanding and utilization of VR interfaces.
(2) Emphasizing user engagement and emotional investment in VR experiences by stimulating interest, curiosity, and immersion through storytelling, gamification, and social interactions. Multisensory interactions that combine visual, auditory, and tactile modalities can further enhance engagement and immersion.
(3) Focusing on user perception and interaction within the virtual world to enhance satisfaction through intuitive controls, feedback, and interactions. Personalization and adaptive design adjustments based on user behavior, preferences, and capabilities can lead to more satisfying VR experiences.

By integrating these theories into VR interaction interface design, continuously evaluating user interactions, and iterating designs based on feedback, we can create more intuitive, engaging, and effective user experiences. This will foster a broader application of VR technology across educational, entertainment, and professional fields.

### 4.2. VR Interactive Interface Evaluation Methods

This review found that empirical evaluation methods are widely used in VR interaction interface design, as evidenced by a review of relevant literature and findings. These methods involve measuring both objective and subjective factors of participants completing designated VR interaction tasks to compare different design schemes. For instance, Sawyer et al. [91] investigated enhancing readability of text on complex backgrounds in VR by manipulating variables such as transparency, blur, color, and font weight, with 124 users participating. Vosinakis and Koutsabasis [16] tested 32 users across 1280 trials, revealing that object highlighting and shading were more effective than wireframe designs in VR gesture interfaces using desktop or HMD setups. Viciana-Abad et al. [24] compared eight combinations of visual, auditory, and force feedback designs based on task performance and presence evaluations among 32 users in gaming scenarios. Control over research environments and manipulation of research variables enhance the applicability and generalizability of conclusions. However, methods that directly obtain feedback from users may be limited by sample size and user bias. We found that some experimental studies have a limited number of participants, which may constrain the generalizability and applicability of their findings or fail to detect actual differences or effects present while also being more susceptible to various biases. Caution should be exercised when interpreting the results of user studies with small sample sizes, and future research should seek to verify these findings through larger-sample studies to ensure that the results are replicable and generalizable to a broader range of users. Furthermore, incorporating qualitative research methods can provide a more comprehensive understanding of the complexities of user experiences in VR interactions.

Analytical evaluation methods, which assess VR interaction interfaces through theoretical analysis and model construction, can provide guidance during the design phase. These methods typically involve observing user behavior, researching interaction experiences, and interviewing participants for feedback [92]. Conducting analytical evaluations in the early stages of design helps in understanding users’ mental models of interface use, evaluating core needs, and exploring potential design spaces. For example, Quesnel and Riecke [93] investigated how VR interaction interface design evokes a sense of awe by initially collecting participants’ introspective interviews and self-report questionnaires on awe experiences. Wang et al. [94] performed factor analysis on 250 surveys to develop an evaluation index system for VR reading experiences. Gao et al. [95] developed a questionnaire to assess the quality of interactions with objects in AR/VR worlds through a combination of literature review and interviews. Nonetheless, the utilization of analytical evaluations is somewhat limited, primarily owing to the lack of established VR interaction interface design guidelines. Furthermore, predictions about user behavior may be subject to inaccuracy due to the absence of empirical validation through practical application.

Some studies integrate the advantages of both empirical and analytical approaches. They begin with analytical evaluations to guide experimental design, selecting focused and effective measurement methods. Subsequently, empirical evaluations validate the impact of design schemes on interaction experiences. Finally, surveys and interviews provide clarity and depth to the research findings, culminating in a compilation of insightful suggestions for the design of VR interaction interfaces [8,10,22,31,96,97]. Mixed methods provide a more holistic view. However, the design and analysis processes are more complex, necessitating more time and resources.

In summary, researchers should consider the research objectives and the availability of resources when selecting evaluation methods and recognize the potential limitations inherent in each approach. Enhancing the reliability and validity of research outcomes can be achieved by integrating a variety of assessment methods. This review summarized the commonly used evaluation content and metrics in the literature (Table 2). Evaluation focuses primarily on user performance in completing interaction tasks through the interface, physiological data related to task stress, user perception of the VR environment, and individual feelings and attitudes. Objective and subjective evaluation data collected can be analyzed using statistical methods to provide specific design recommendations.

Objective evaluation of VR interaction interface design commonly measures task completion time, accuracy, and errors. Additionally, some studies meticulously observe and document user behaviors, including pauses, requests for assistance, revisiting steps, unnecessary actions, and looking away during task execution. These observations can provide valuable insights into the intuitiveness and user-friendliness of the interface. However, the interpretations of these behaviors may vary among researchers. It is also important to recognize that these metrics may not fully capture the user experience and can be affected by factors beyond interface design, such as individual differences in user proficiency, task familiarity, age, gender, or intelligence. Using sensors or tracking devices to capture users’ physiological signals can objectively reflect the interaction experience. Currently used physiological indicators include blood pressure, heart rate, body temperature, electroencephalogram (EEG), electrooculogram (EOG), electrocardiogram (ECG), galvanic skin response (GSR), and eye blink rate (EBR). Mean respiratory rate, respiratory rate variability, and skin temperature can be used to estimate cognitive workload in haptic interaction [45]. Providing real-time feedback of measured physiological data to the VR system helps enhance the interaction experience, such as designing VR game interfaces adapted to players’ emotional states using EEG data [98], optimizing visual element layout in VR interaction interfaces based on users’ gaze duration and fixation points analyzed with eye trackers [99], and adjusting the virtual fitness system’s exercise scene interface visual elements based on user heart rate and scientific fitness prescription to prompt users to alter their exercise strategies [5]. While physiological measurements provide objective data for assessing user stress and sense of immersion, they can be affected by environmental factors and individual differences. Additionally, the cost and complexity of the required equipment may restrict their widespread adoption and ease of implementation. In addition, some studies evaluate users’ spatial perception of virtual environments, involving understanding of spatial attributes and relationships such as depth, shape, and size ratios [15,100]. Sorger, J. et al. [31] measured indicators such as angle deviation, path deviation, accuracy in judging spatial relationships, and navigation speed in an abstract three-dimensional data space to assess three head-mounted display (HMD)-based VR interaction interface designs. Individual differences in spatial cognition can affect the reliability of such evaluations. While a sample of participants who are homogeneous in terms of spatial cognitive abilities can yield more consistent and predictable results, the homogeneity of study samples may limit the relevance of the findings to a wider user base, as the study may not capture the full spectrum of variability present in the general population. Researchers must meticulously consider the composition of their samples, striving for a balance between homogeneity and heterogeneity.

Subjective evaluation of VR interaction interface design aims to understand potential users’ or domain experts’ attitudes toward design proposals, as well as their psychological and bodily experiences related to tasks or environments. Common subjective evaluation indicators include usability, satisfaction, preferences, sense of presence (SOP), sense of agency (SOA), and simulator sickness, with respondents providing feedback in the form of answers or ratings. For example, research by Zhang et al. [11] conducted a formal user study to evaluate the usability of the hybrid interface, utilizing six user experience questionnaire scales. Sun, L. et al. [61] evaluated the usability, satisfaction, and learning experience of an immersive VR museum’s video interaction interface to compare different information presentation formats. Kim M. et al. [23] explored head-mounted display-based gaze interaction interface designs from four aspects—field of view, feedback format, information dimension, and background color. After 35 users completed selection tasks using 12 design scenarios, they filled out multidimensional questionnaires on SOP and satisfaction. Some subjective scales with high reliability and validity have been widely used by scholars, such as the System Usability Scale (SUS), NASA Task Load Index (NASA TLX), Simulator Sickness Questionnaire (SSQ), Questionnaire for User Interaction Satisfaction (QUIS), Technology Acceptance Model (TAM), User Experience Questionnaire (UEQ), After-Scenario Questionnaire (ASQ), and Game Experience Questionnaire (GEQ). Utilizing standardized evaluation tools such as established questionnaires and rating scales can provide a structured approach to gathering user feedback. It’s important to select tools that have been validated for use in similar contexts to ensure they are sensitive to the nuances of VR experiences. However, these measures are inherently subjective and may be influenced by user expectations, individual differences, and the contextual setting of the evaluation. To counteract the effects of individual biases and gain a more comprehensive understanding of user attitudes and experiences, it is essential to diversify the participant pool to include individuals with varied backgrounds, experiences, and expectations. Furthermore, providing participants with clear and unbiased instructions aligns their expectations with the goals of the evaluation and minimizes the risk of their responses being influenced by preconceived notions about the VR interface. Discussing the potential impact of subjective evaluation limitations on the findings and the generalizability of the results is crucial for allowing readers to critically assess the study. By employing these strategies, researchers can enhance the reliability and validity of subjective evaluations, resulting in a more profound and nuanced comprehension of user experiences in VR interaction interface design.

The design and evaluation criteria vary depending on the research questions. For VR applications in scenarios such as reading, gaming, and shopping, objective task performance is not always the primary focus. Therefore, Wang et al. [65], Benlamine et al. [98], Roupe et al. [100], and others have conducted subjective evaluations of VR interaction interfaces. Similarly, Su et al. [96] integrated quantitative and qualitative methods, using Sound Thinking, QUIS, UEQ, and focus group interviews to understand shopping experiences based on HMD-based VR interaction interfaces. Shafer [101] utilized SSQ, GEQ, and scales assessing SOP, interactivity, realism, and enjoyment to explore the impact of interface fidelity on HMD-based VR gaming experiences. In Grabowski’s study [9], 87 users completed SSQ, TAM, SUS, and surveys on SOP, stress, and training evaluation after immersive VR firefighting training.

More studies have conducted comprehensive evaluations of VR interaction interface design from both subjective and objective perspectives. After measuring objective performance metrics such as task completion time, accuracy, and error rates, various studies have proceeded with subjective evaluations. For instance, Wang et al. [8] asked users to rate the usability, learnability, efficiency, accuracy, and preferences of VR menu interfaces and to complete NASA TLX. Zhao and Allison [52] assessed user preferences for binary responses in virtual environments via a questionnaire, comparing a head gesture interface, a hand gesture interface, and a conventional gamepad interface. Jeong et al. [10] had users rate discomfort and visual fatigue for nine immersive VR menu interfaces. Vosinakis and Koutsabasis [16] collected satisfaction questionnaires for five gesture-based interaction visual feedback designs. Coelho et al. [18] used SUS, ASQ, and scales related to SOP to evaluate three VR video creation interfaces. Ziadeh et al. [22] collected ratings from 22 users on presence, ownership, frustration, and embodiment in a study on brain-computer interface-based virtual avatars. Sorger et al. [31] asked users to select preferred VR interaction interfaces and complete SSQ and NASA TLX. Lou et al. [102] assessed the position of VR gesture interaction interfaces using SUS, NASA TLX, and the Borg15 scale for perceived physical exertion. Hinricher et al. [81] assessed how design factors of VR control elements impact users’ sense of presence and mental workload. Among them, several studies combined semi-structured interviews after user testing and questionnaires to elicit users’ explanations of their experiences and attitudes [8,10,22,31,96].

We have found a scarcity of studies conducting long-duration user studies. As users’ adaptation to VR evolves over time and with accumulated experience, ongoing user studies are essential to ensure the accuracy and reliability of assessments concerning user satisfaction and performance in VR applications. Long-duration user studies help developers understand how users’ behavioral patterns, interaction habits, and preferences for interfaces evolve, thereby informing interface design. For instance, it can guide the optimization of interaction design to reduce the learning curve for users or the introduction of novel elements to sustain engagement. In summary, longitudinal study is crucial for the design of VR interaction interfaces.

## 5. Conclusions and Perspectives

VR interaction interfaces are crucial for providing users with a positive experience. This review surveyed a total of 438 articles on VR interaction interface design retrieved from the Web of Science and CNKI databases. The aim was to explore global research hotspots and emerging topics, as well as to anticipate future key issues and directions in VR interaction interface design.

Since 2016, there has been a steady increase in the number of publications on VR interaction interface design, with a majority featured in computer science journals. However, there is notably less attention from the humanities disciplines towards VR interaction interfaces. The high-frequency keywords in the literature indicate a significant trend towards multi-channel interaction design. The majority of research topics in the related literature focus on specific VR application scenarios, while fewer studies have been dedicated to design evaluation and the proposal of design methods. Evaluation methods for VR interaction interface design primarily emphasize empirical approaches, with few employing analytical evaluations.

Our review has effectively addressed significant gaps in the literature by synthesizing a wide range of studies and providing a thorough evaluation of VR interface design principles and evaluation methods. By integrating diverse perspectives and methodologies, we have highlighted the need for further research in design evaluation. Investigating adaptive interfaces that respond to real-time user feedback or conducting longitudinal studies to assess user adaptation over time could prove valuable. Understanding the impact of different VR interaction interface designs on both user objective performance and subjective experiences is crucial for refining the development of standards and guidelines for VR interactive interface design.

This review identified the following key areas for future research in VR interaction interface design:

(1) A multi-channel VR interaction interface that conveys information in a manner consistent with human cognitive and emotional processes can enhance immersion and interaction depth, leading to more intuitive and satisfying user experiences and thus fostering innovation in VR interaction interface design.
(2) Focusing on the diverse needs of users is not only crucial for enhancing the inclusiveness of VR but also aligns with Sustainable Development Goal (SDG) 10, which aims to reduce inequalities. By providing inclusive VR interaction interfaces, we can reduce disparities in technology usage and promote equitable access to information and resources.
(3) Interdisciplinary approaches that blend psychology, design, and technology are crucial for driving innovation in VR interaction interfaces. The integration of these fields helps to create personalized and adaptive interfaces, which can enhance user engagement and satisfaction.

In conclusion, our review has not only summarized the existing design principles and evaluation methods but has also provided a critical analysis of the current literature, identifying gaps and providing a foundation for future research endeavors. Due to space and literature source constraints, this review focuses on academic journal articles from 2011 to 2023, summarizing research focuses and key findings. Future work could analyze the latest conference presentations and engage in deeper theoretical discussions. With the continuous advancement of VR technology and related research, the potential applications of VR interaction interfaces in various domains are expanding. The innovation in VR interaction interfaces is not only about technological advancement but also about understanding how these technologies can be harnessed to meet user needs and expectations. We are confident that the findings of this review will significantly aid researchers and practitioners in advancing the frontiers of VR interaction interface design.

## Figures and Tables

**Table 1:** Journals with more literature on VR interaction interface design.

Journal	Number	Publisher City
1. Virtual Reality	25	London, UK
2. IEEE Transactions on Visualization and Computer Graphics	20	Los Alamitos, NM, USA
3. Applied Sciences—Basel	17	Basel, Switzerland
4. IEEE Access	15	Piscataway, NJ, USA
5. Multimedia Tools and Applications	15	Dordrecht, Netherlands
6. Computer Animation and Virtual Worlds	12	Los Alamitos, NM, USA
7. Computers & Education	10	Oxford, UK
8. Packaging Engineering	10	Chongqing, China
9. Symmetry-Basel	9	Basel, Switzerland
10. International Journal of Human-Computer Studies	9	London, UK
11. Computers & Graphics-UK	9	Oxford, UK
12. Sustainability	7	Basel, Switzerland
13. Research and Exploration in Laboratory	6	Shanghai, China
14. Sensors	5	Basel, Switzerland
15. IEEE Transactions on Haptics	5	Los Alamitos, NM, USA
16. Journal of Neuroengineering and Rehabilitation	5	London, UK
17. Frontiers in Psychology	4	Lausanne, Switzerland
18. Computers in Human Behavior	4	Oxford, UK
19. Ship Science and Technology	4	Beijing, China
20. Behaviour & Information Technology	4	Abingdon, UK

**Table 2:** A summary of the evaluation of VR interaction interface design.

Evaluation Content	Type	Metrics
Task performance	objective	Task completion time, accuracy rate, number of errors, pause time, number of help requests, etc.
Physiological measures	objective	Blood pressure, heart rate, body temperature, respiratory rate, EEG, EOG, ECG, GSR, EBR, etc.
Environmental perception	objective	Accuracy in judging spatial attributes and relationships, angle deviation, and path deviation
Feelings and attitudes	subjective	Positive aspects: usability, satisfaction, preference, presence, agency, ease of use, utility, interactivity, realism, enjoyment, learnability, efficiency, accuracy, ownership, and embodiment. Negative aspects: simulation sickness, task load, stress, fatigue, discomfort, visual fatigue, and frustration.

## References

[1] Rebelo F., Noriega P., Duarte E., Soares M. (2012). Using virtual reality to assess user experience. Hum. Factors.

[2] Burdea G.C., Coiffet P. (2003). Virtual Reality Technology.

[3] Bekele M.K., Pierdicca R., Frontoni E., Malinverni E.S., Gain J. (2018). A survey of augmented, virtual, and mixed reality for cultural heritage. ACM J. Comput. Cult. Herit..

[4] Chen C.-H., Chen M.-X. (2020). Effects of the design of overview maps on three-dimensional virtual environment interfaces. Sensors.

[5] Zhao H., Meng Q., Han L., Pan Z. (2015). Design and implementation of the personalized virtual fitness system. J. Image Graph..

[6] Sudár A., Csapó A.B. (2023). Descriptive markers for the cognitive profiling of desktop 3D spaces. Electronics.

[7] Andujar C., Chica A., Brunet P. (2012). User-interface design for the Ripoll Monastery exhibition at the National Art Museum of Catalonia. Comput. Graph..

[8] Wang Y., Hu Y., Chen Y. (2021). An experimental investigation of menu selection for immersive virtual environments: Fixed versus handheld menus. Virtual Real..

[9] Grabowski A. (2021). Practical skills training in enclosure fires: An experimental study with cadets and firefighters using CAVE and HMD-based virtual training simulators. Fire Saf. J..

[10] Jeong S., Jung E.S., Im Y. (2016). Ergonomic evaluation of interaction techniques and 3D menus for the practical design of 3D stereoscopic displays. Int. J. Ind. Ergon..

[11] Zhang L., He W., Bai H., Zou Q., Wang S., Billinghurst M. (2023). A hybrid 2D-3D tangible interface combining a smartphone and controller for virtual reality. Virtual Real..

[12] Nezami F.N., Wachter M.A., Maleki N., Spaniol P., Kuhne L.M., Haas A., Pingel J.M., Tiemann L., Nienhaus F., Keller L. (2021). Westdrive X LoopAR: An open-access virtual reality project in Unity for evaluating user interaction methods during takeover requests. Sensors.

[13] Molina G., Gimeno J., Portalés C., Casas S. (2022). A comparative analysis of two immersive virtual reality systems in the integration and visualization of natural hand interaction. Multimed. Tools Appl..

[14] Lee Y.S., Sohn B.S. (2018). Immersive gesture interfaces for navigation of 3D maps in HMD-based mobile virtual environments. Mob. Inf. Syst..

[15] Lukacevic F., Skec S., Perisic M.M., Horvat N., Storga M. (2020). Spatial perception of 3D CAD model dimensions and affordances in virtual environments. IEEE Access.

[16] Vosinakis S., Koutsabasis P. (2018). Evaluation of visual feedback techniques for virtual grasping with bare hands using Leap Motion and Oculus Rift. Virtual Real..

[17] Wagner J.A., Stuerzlinger W., Nedel L. (2020). Evaluating an immersive space-time cube geovisualization for intuitive trajectory data exploration. IEEE Trans. Visual. Comput. Graph..

[18] Coelho H., Melo M., Barbosa L., Martins J., Teixeira M.S., Bessa M. (2019). Authoring tools for creating 360 multisensory videos-Evaluation of different interfaces. Expert Syst..

[19] Fengjun Z., Guozhong D., Xiaolan P. (2016). A survey on human-computer interaction in virtual reality. Sci. China Ser. F-Inf. Sci..

[20] Huang J., Han D., Chen Y., Tian F., Wang H., Dai G. (2016). A survey on human-computer interaction in mixed reality. J. Comput.-Aided Des. Comput. Graph..

[21] Bowman D.A., Kruijff E., LaViola J.J., Poupyrev I. (2004). 3D User Interfaces: Theory and Practice.

[22] Ziadeh H., Gulyas D., Nielsen L.D., Lehmann S., Nielsen T.B., Kjeldsen T.K.K., Hougaard B.I., Jochumsen M., Knoche H. (2021). “Mine works better”: Examining the influence of embodiment in virtual reality on the sense of agency during a binary motor imagery task with a brain-computer interface. Front. Psychol..

[23] Kim M., Lee J., Jeon C., Kim J. (2017). A study on interaction of gaze pointer-based user interface in mobile virtual reality environment. Symmetry.

[24] Viciana-Abad R., Reyes-Lecuona A., Rosa-Pujazon A., Perez-Lorenzo J.M. (2014). The influence of different sensory cues as selection feedback and co-location in presence and task performance. Multimed. Tools Appl..

[25] Wang Y., Hu Z., Li P., Yao S., Liu H. (2022). Multiple perspectives integration for virtual reality-aided assemblability assessment in narrow assembly spaces. Int. J. Adv. Manuf. Technol..

[26] Kim J., Hwang J.I., Lee J. (2022). VR Color picker: Three-dimensional color selection interfaces. IEEE Access.

[27] Woodworth J.W., Borst C.W. (2023). Pointing in the third-person: An exploration of human motion and visual pointing aids for 3D virtual mirrors. Virtual Real..

[28] Jia D.W., Bhatti A., Nahavandi S., Horan B. (2013). Human performance measures for interactive haptic-audio-visual interfaces. IEEE Trans. Haptic.

[29] Alkemade R., Verbeek F.J., Lukosch S.G. (2017). On the efficiency of a VR hand gesture-based interface for 3D object manipulations in conceptual design. Int. J. Hum.-Comput. Interact..

[30] Lahav O. (2022). Virtual reality systems as an orientation aid for people who are blind to acquire new spatial information. Sensors.

[31] Sorger J., Arleo A., Kan P., Knecht W., Waldner M. (2021). Egocentric network exploration for immersive analytics. Comput. Graph. Forum.

[32] Sun C., Hu W., Xu D. (2019). Navigation modes, operation methods, observation scales and background options in UI design for high learning performance in VR-based architectural applications. J. Comput. Des. Eng..

[33] de Freitas B.L., da Silva T.D., Crocetta T.B., Massetti T., de Araujo L.V., Coe S., Dawes H., Caromano F.A., Monteiro C.B.D. (2019). Analysis of different device interactions in a virtual reality task in individuals with duchenne muscular dystrophy-A randomized controlled trial. Front. Neurol..

[34] Zhou C., Huang T., Liang S. (2021). Smart home R&D system based on virtual reality. J. Intell. Fuzzy Syst..

[35] Kim W., Xiong S. (2022). Pseudo-haptic button for improving user experience of mid-air interaction in VR. Int. J. Hum. Comput. Stud..

[36] Otaran A., Farkhatdinov I. (2022). Haptic ankle platform for interactive walking in virtual reality. IEEE Trans. Visual. Comput. Graph..

[37] Cardoso J.C.S., Ribeiro J.M. (2021). Tangible VR book: Exploring the design space of marker-based tangible interfaces for virtual reality. Appl. Sci..

[38] Butnariu S., Duguleana M., Brondi R., Girbacia F., Postelnicu C., Carrozzino M. (2018). An interactive haptic system for experiencing traditional archery. Acta Polytech. Hung..

[39] Yang T.H., Son H., Byeon S., Gil H., Hwang I., Jo G., Choi S., Kim S.Y., Kim J.R. (2021). Magnetorheological fluid haptic shoes for walking in VR. IEEE Trans. Haptic.

[40] Islam S., Ashour R., Sunda-Meya A. (2019). Haptic and virtual reality based shared control for MAV. IEEE Trans. Aerosp. Electron. Syst..

[41] Gosselin F., Bouchigny S., Megard C., Taha F., Delcampe P., d’Hauthuille C. (2013). Haptic systems for training sensorimotor skills: A use case in surgery. Robot. Auton. Syst..

[42] Ceccacci S., Generosi A., Leopardi A., Mengoni M., Mandorli A.F. (2021). The role of haptic feedback and gamification in virtual museum systems. ACM J. Comput. Cult. Herit..

[43] Jin S.A.A. (2013). The moderating role of sensation seeking tendency in robotic haptic interfaces. Behav. Inform. Technol..

[44] Carlson P., Vance J.M., Berg M. (2016). An evaluation of asymmetric interfaces for bimanual virtual assembly with haptics. Virtual Real..

[45] Novak D., Mihelj M., Munih M. (2011). Psychophysiological responses to different levels of cognitive and physical workload in haptic interaction. Robotica.

[46] Reynaert V., Rekik Y., Berthaut F., Grisoni L. (2023). The effect of hands synchronicity on users perceived arms Fatigue in Virtual reality environment. Int. J. Hum. Comput. Stud..

[47] Park K.B., Lee J.Y. (2019). New design and comparative analysis of smartwatch metaphor-based hand gestures for 3D navigation in mobile virtual reality. Multimed. Tools Appl..

[48] Zhang S., Liu Y., Song F., Yu D., Bo Z., Zhang Z. (2023). The effect of audiovisual spatial design on user experience of bare-hand interaction in VR. Int. J. Hum.-Comput. Interact..

[49] De Paolis L.T., De Luca V. (2022). The effects of touchless interaction on usability and sense of presence in a virtual environment. Virtual Real..

[50] Yeh S.C., Wu E.H.K., Lee Y.R., Vaitheeshwari R., Chang C.W. (2022). User experience of virtual-reality interactive interfaces: A comparison between hand gesture recognition and joystick control for XRSPACE MANOVA. Appl. Sci..

[51] Wang P., Zhang S.S., Bai X.L., Billinghurst M., He W.P., Sun M.M., Chen Y.X., Lv H., Ji H.Y. (2019). 2.5DHANDS: A gesture-based MR remote collaborative platform. Int. J. Adv. Manuf. Technol..

[52] Zhao J.B., Allison R.S. (2020). Comparing head gesture, hand gesture and gamepad interfaces for answering Yes/No questions in virtual environments. Virtual Real..

[53] Mashal S., Kranz M., Hoelzl G. (2020). Do you feel like flying? A study of flying perception in virtual reality for future game development. IEEE Comput. Graph. Appl..

[54] Valdivia S., Blanco R., Uribe-Quevedo A., Penuela L., Rojas D., Kapralos B. (2018). Development and evaluation of two posture-tracking user interfaces for occupational health care. Adv. Mech. Eng..

[55] Zari G., Condino S., Cutolo F., Ferrari V. (2023). Magic Leap 1 versus Microsoft HoloLens 2 for the visualization of 3D content obtained from radiological images. Sensors.

[56] Lu F., Nanjappan V., Parsons P., Yu L., Liang H.-N. (2022). Effect of display platforms on spatial knowledge acquisition and engagement: An evaluation with 3D geometry visualizations. J. Vis..

[57] Wang Y., Wang X., Han L. (2023). Research on Great Wall section protection and user VR experience innovation based on GIS data visualization. Soft Comput..

[58] Zhang H., Zhu L., Zhang Q., Wang Y., Song A. (2023). Online view enhancement for exploration inside medical volumetric data using virtual reality. Comput. Biol. Med..

[59] Xu H., Zhang J. (2021). Large relics scenario-based visualization using head-mounted displays. Comput. Intell. Neurosci..

[60] Bardella F., Rodrigues A.M., Neto R.M.L. (2019). Crystalwalk: An educational interactive software for synthesis and visualization of crystal structures. J. Mater. Educ..

[61] Sun H., Lv J., Cun W. (2018). VR system information visualization model cognition. J. Graph..

[62] Zwolinski G., Kaminska D., Laska-Lesniewicz A., Haamer R.E., Vairinhos M., Raposo R., Urem F., Reisinho P. (2022). Extended reality in education and training: Case studies in management education. Electronics.

[63] Cho Y., Park M., Kim J. (2023). XAVE: Cross-platform based asymmetric virtual environment for immersive content. IEEE Access.

[64] Zhu Y., Lou Z., Wu T., Wang J. (2023). Research on system of virtual-reality fusion and inquiry-based learning. J. Syst. Simul..

[65] Wang P., Bai X.L., Billinghurst M., Zhang S.S., Han D.C., Sun M.M., Wang Z., Lv H., Han S. (2020). Haptic feedback helps me? A VR-SAR remote collaborative system with tangible interaction. Int. J. Hum.-Comput. Interact..

[66] Choi Y., Kim Y.S. (2022). An adaptive UI based on user-satisfaction prediction in mixed reality. Appl. Sci..

[67] Shankhwar K., Smith S. (2022). An interactive extended reality-based tutorial system for fundamental manual metal arc welding training. Virtual Real..

[68] Cong X., Li T. (2020). Design and development of virtual medical system interface based on VR-AR hybrid technology. Comput. Math. Methods. Med..

[69] Tsao Y.-C., Shu C.-C., Lan T.-S. (2019). Development of a reminiscence therapy system for the elderly using the integration of virtual reality and augmented reality. Sustainability.

[70] Anton D., Kurillo G., Bajcsy R. (2018). User experience and interaction performance in 2D/3D telecollaboration. Future Gener. Comput. Syst..

[71] Lo C.-M., Wang J.-H., Wang H.-W. (2022). Virtual reality human-robot interaction technology acceptance model for learning direct current and alternating current. J. Supercomput..

[72] Yu S., Liu Q., Johnson-Glenberg M.C., Han M., Ma J., Ba S., Wu L. (2023). Promoting musical instrument learning in virtual reality environment: Effects of embodiment and visual cues. Comput. Educ..

[73] Hulusic V., Gusia L., Luci N., Smith M. (2023). Tangible user interfaces for enhancing user experience of virtual reality cultural heritage applications for utilization in educational environment. ACM J. Comput. Cult. Herit..

[74] Al Hakim V.G., Yang S.-H., Liyanawatta M., Wang J.-H., Chen G.-D. (2022). Robots in situated learning classrooms with immediate feedback mechanisms to improve students’ learning performance. Comput. Educ..

[75] Roldan J.J., Pena-Tapia E., Martin-Barrio A., Olivares-Mendez M.A., Del Cerro J., Barrientos A. (2017). Multi-robot interfaces and operator situational awareness: Study of the impact of immersion and prediction. Sensors.

[76] Dai M., Li L., Lu Y., Xiao L., Zong X., Tu C., Meng F., Tang Y., Guo D. (2023). Research on holographic visualization verification platform for construction machinery based on mixed reality technology. Appl. Sci..

[77] Lee H.Y., Zhou P., Duan A.Q., Wang J.L., Wu V.C., Navarro-Alarcon D. (2023). A multisensor interface to improve the learning experience in arc welding taining tasks. IEEE Trans. Hum.-Mach. Syst..

[78] Prati E., Villani V., Peruzzini M., Sabattini L. (2021). An approach based on VR to design industrial human-robot collaborative workstations. Appl. Sci..

[79] de Lotbiniere-Bassett M., Batista A.V., Lai C., El Chemaly T., Dort J., Blevins N., Lui J. (2023). The user experience design of a novel microscope within SurgiSim, a virtual reality surgical simulator. Int. J. Comput. Assist. Radiol. Surg..

[80] Vourvopoulos A., Badia S.B.I. (2016). Motor priming in virtual reality can augment motor-imagery training efficacy in restorative brain-computer interaction: A within-subject analysis. J. Neuroeng. Rehabil..

[81] Hinricher N., Schröer C., Backhaus C. (2023). Design of control elements in virtual reality-investigation of factors influencing operating efficiency, user experience, presence, and workload. Appl. Sci..

[82] Weidner F., Maier J.E., Broll W. (2023). Eating, smelling, and seeing: Investigating multisensory integration and (in)congruent stimuli while eating in VR. IEEE Trans. Visual. Comput. Graph..

[83] Han D.I.D., Melissen F., Haggis-Burridge M. (2023). Immersive experience framework: A Delphi approach. Behav. Inform. Technol..

[84] Zhang W., Min W. (2018). Characteristics of virtual user interface for teenagers. Packag. Eng..

[85] Li X., Zhao F., Zhang S., Zhang L., Xu M. (2018). Research on the design and application of VR/AR learning experience. China Educ. Technol..

[86] Qi B., Zhang W., Zhu X., Zhang M., Li Q. (2023). Research on multi-mode interaction model and influencing factors for digital collections. Libr. Inf. Serv..

[87] Lehrer N., Attygalle S., Wolf S.L., Rikakis T. (2011). Exploring the bases for a mixed reality stroke rehabilitation system, Part I: A unified approach for representing action, quantitative evaluation, and interactive feedback. J. Neuroeng. Rehabil..

[88] Jacob R.J.K., Girouard A., Hirshfield L.M., Horn M.S., Shaer O., Solovey E.T., Zigelbaum J. (2008). Reality-based interaction: A framework for post-WIMP interfaces. Proceedings of the SIGCHI Conference on Human Factors in Computing Systems.

[89] Wang Y., Zhai G., Chen S., Min X., Gao Z., Song X. (2019). Assessment of eye fatigue caused by head-mounted displays using eye-tracking. Biomed. Eng. Online.

[90] Lin Y., Chen J., Liu Y., Wang Y. (2015). User experience design of VR-AR hybrid mobile browsing system based on mental model. Chin. J. Comput..

[91] Sawyer B., Wolfe B., Dobres J., Chahine N., Mehler B., Reimer B. (2020). Glanceable, legible typography over complex backgrounds. Ergonomics.

[92] Wright V., Kefalidou G. (2022). Can you hear the colour? Designing virtual worlds for synaesthetic and multimodal experiences. Interact. Comput..

[93] Quesnel D., Riecke B.E. (2018). Are you awed yet? How virtual reality gives us awe and goose bumps. Front. Psychol..

[94] Wang X., Zheng G., Wang D., Tao B. (2020). Evaluation and empirical study of virtual reality interactive reading experience from the perspective of human-computer interaction. Libr. Inf. Serv..

[95] Gao M.Y.Z., Boehm-Davis D.A. (2023). Development of a customizable interactions questionnaire (CIQ) for evaluating interactions with objects in augmented/virtual reality. Virtual Real..

[96] Su K.-W., Chen S.-C., Lin P.-H., Hsieh C.-I. (2020). Evaluating the user interface and experience of VR in the electronic commerce environment: A hybrid approach. Virtual Real..

[97] Wang J., Lindeman R. (2015). Coordinated hybrid virtual environments: Seamless interaction contexts for effective virtual reality. Comput. Graph..

[98] Benlamine M.S., Dufresne A., Beauchamp M.H., Frasson C. (2021). BARGAIN: Behavioral affective rule-based games adaptation interface-towards emotionally intelligent games: Application on a virtual reality environment for socio-moral development. User Model. User-Adapt. Interact..

[99] Sun L., Zhou Y., Hansen P., Geng W., Li X. (2018). Cross-objects user interfaces for video interaction in virtual reality museum context. Multimed. Tools Appl..

[100] Roupe M., Bosch-Sijtsema P., Johansson M. (2014). Interactive navigation interface for virtual reality using the human body. Comput. Environ. Urban. Syst..

[101] Shafer D.M. (2021). The Effects of interaction fidelity on game experience in virtual reality. Psychol. Pop. Media.

[102] Lou X., Li X., Hansen P., Du P. (2021). Hand-adaptive user interface: Improved gestural interaction in virtual reality. Virtual Real..

